# Straightforward Approach for Preparing Durable Antibacterial ZnO Nanoparticle Coatings on Flexible Substrates

**DOI:** 10.3390/molecules27227672

**Published:** 2022-11-08

**Authors:** Andris Šutka, Linda Mežule, Viktorija Denisova, Jochen Meier-Haack, Akshay Kulkarni, Sanda Bitina, Krisjanis Smits, Svetlana Vihodceva

**Affiliations:** 1 Institute of Materials and Surface Engineering, Faculty of Materials Science and Applied Chemistry, Riga Technical University, P. Valdena 3, LV-1048 Riga, Latvia; 2 Water Research and Environmental Biotehnology Laboratory, Faculty of Civil Engineering, Riga Technical University, P. Valdena 1, LV-1048 Riga, Latvia; 3 Polymeric Membrane Materials Group, Department Processing Technology, Leibniz-Institut fuer Polymerforschung Dresden e.V., Hohe Straße 6, 01069 Dresden, Germany; 4 Institute of Solid State Physics, University of Latvia, Kengaraga 8, LV-1063 Riga, Latvia

**Keywords:** ZnO, nanoparticle, coating, flexible substrate, antibacterial, *E. coli*, *S. aureus*

## Abstract

Flexible antibacterial materials have gained utmost importance in protection from the distribution of bacteria and viruses due to the exceptional variety of applications. Herein, we demonstrate a readily scalable and rapid single-step approach for producing durable ZnO nanoparticle antibacterial coating on flexible polymer substrates at room temperature. Substrates used are polystyrene, poly(ethylene-co-vinyl acetate) copolymer, poly(methyl methacrylate), polypropylene, high density polyethylene and a commercial acrylate type adhesive tape. The deposition was achieved by a spin-coating process using a slurry of ZnO nanoparticles in toluene. A stable modification layer was obtained when toluene was a solvent for the polymer substrates, namely polystyrene and poly(ethylene-co-vinyl acetate). These coatings show high antibacterial efficiency causing >5 log decrease in the viable counts of Gram-negative bacteria *Escherichia. coli* and Gram-positive bacteria *Staphylococcus aureus* in 120 min. Even after tapping these coated surfaces 500 times, the antibacterial properties remained unchanged, showing that the coating obtained by the presented method is very robust. In contrast to the above findings, the coatings are unstable when toluene is not a solvent for the substrate.

## 1. Introduction

Nano-sized ZnO has been recognized as a promising antibacterial material, showing significant elimination of activity for a wide spectrum of bacterial species [1,2,3,4]. The antibacterial activity of ZnO nanoparticles is attributed to the release of Zn^2+^ ions, as well as superoxide radicals in the dark [5,6,7,8,9]. The antibacterial activity of ZnO can be further enhanced by UV light irradiation due to photocatalytic generation of reactive oxygen species by reaction with water and oxygen [10,11]. ZnO is an inexpensive material and nanoparticles can be synthesized using various chemical and physical synthesis methods [9].

The most challenging task is obtaining durable ZnO nanoparticle coatings onto flexible substrates to apply to surfaces with different shapes. Contrary to rigid inorganic substrates, soft materials cannot tolerate high annealing temperatures, which are needed to transform Zn precursors into crystalline and durable ZnO coatings. To prepare flexible antibacterial material, commonly, ZnO nanoparticles are incorporated into a polymer matrix. However, when nanoparticles are mixed into a polymer matrix, nanoparticle surfaces become passivated by the polymer matrix, leading to significant reduction in antibacterial activity, as the nanoparticles become isolated from microorganisms reaching the surface [12]. Furthermore, substantial amounts of nanoparticles must be used to have a sufficient number of particles on the surface. Since a polymer skin covers the nanoparticles, this must be removed by a post-treatment process, such as plasma etching [12]. 

In addition, other methods are used to obtain durable nanostructured ZnO coatings on flexible substrates. ZnO coatings have been grown by physical and chemical vapor deposition, as well as solution techniques [7,13,14,15,16]. Magnetron sputtering has been used for depositing ZnO nanoparticles on polylactide [8,17]. ZnO nanostructures were deposited on a polyethylene terephthalate sheet and cotton fabric by activated reactive evaporation, where nanostructures are grown in the gas phase and deposited as a nanostructured film at room temperature on the substrates placed above the plasma source [18]. The atomic layer deposition process, which is based on the sequential use of a gas-phase chemical reaction has been used for depositing ZnO on cellulose fibers [19] and silicone rubber [20]. Sol-gel solution approach has been used for coating ZnO films on different substrates at room temperature [21,22]. For example, ZnO has been deposited on polystyrene by sol-gel process [23]. Another effective approach for growing ZnO on flexible substrates is chemical bath deposition, which has been used for depositing a ZnO coating on polypropylene [24]. However, the requirement for seed layers, long processing times, expensive and energetically costly equipment, as well as limited scalability of the approach are the general drawbacks of the proposed deposition techniques. 

The alternative, and more straightforward strategy, is to deposit films from nanoparticle powders by using a small addition of organic binders. ZnO nanoparticle powders can be produced on a large scale by numerous synthesis approaches, thus making this way especially attractive. Agrawal et al. [25] prepared ZnO coated robust antibacterial cotton fabrics by using aminopropyltriethoxysilane to anchor ZnO nanoparticles on the surface of a cotton fabric, followed by modification with a hexadecyltrimethoxysilane as a top protecting hydrophobic layer. This dual-silanization approach resulted in highly durable coatings, presenting good antibacterial properties [25]. ZnO nanoparticles adhered by double silanization remained on the surface at the same level after 2 h of ultrasonic washing. In another study, multilayer nanocomposite films with a high content of ZnO were fabricated on cationized woven cotton fabrics via layer-by-layer molecular self-assembly technique [26]. Functional antibacterial properties of ZnO nanoparticles were achieved by depositing 10 layers. However, both approaches require sequential immersion into the different processing solutions (deposition and washing), thus making production time consuming.

Herein we report a one-cycle rapid deposition of durable ZnO nanoparticle coatings on flexible polymer substrate from ZnO nanoparticle slurries. We show that ZnO nanoparticles from slurries based on organic solvents adhere strongly to polymer substrates, as the polymer is soluble in the chosen solvent. In our approach, antibacterial ZnO nanoparticles are adhered to the polymer surface and are not covered by the polymer matrix, thus providing high antibacterial activity. Coatings show good stability and high antibacterial activity. Among other substrates, we have used the proposed approach to deposit ZnO nanoparticles on flexible commercial adhesive tape, which can be applied to any surface with different shapes and thus has immense potential for a wide range of applications.

## 2. Results and Discussion

### 2.1. Characterization of ZnO Nanoparticles and ZnO Nanoparticle Coatings

**Figure 1:**
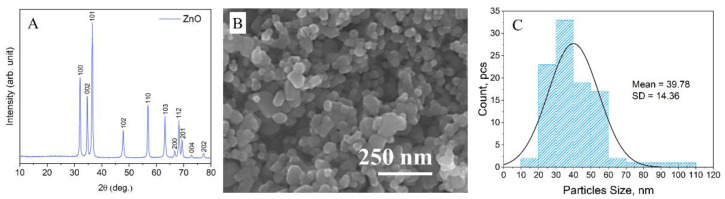
(**A**) XRD pattern of commercial ZnO nanoparticles, (**B**) SEM image of commercial ZnO nanoparticles used in the preparation of coatings, and (**C**) the ZnO nanoparticle size distribution histogram, with normal distribution curve, the mean value and standard deviation (SD) are reported.

XRD and SEM were used to characterize the ZnO nanoparticles. The powder diffractogram of the ZnO particles (Figure 1A) shows the typical pattern for a wurtzite-like (hexagonal) crystal structure and is in good agreement with PDF#04-005-5076. Furthermore, the intense narrow diffraction peaks indicate a high crystallinity. 

The ZnO particles predominantly have a spherical shape with a diameter of roughly 40 nm, as observed in the SEM image (Figure 1B). However, there are also some particles with a plate-like structure and a much larger size. A nanoparticle size distribution histogram determined from the SEM images by measuring the diameter of 100 random nanoparticles using ImageJ software showed a significant variation in size ranging from ~20 to ~110 nm, with an average size of ~40 nm (Figure 1C).

ZnO nanoparticles in a toluene dispersion were deposited by spin-coating onto the polymer substrates having different solubility in toluene. The substrates chosen were polystyrene (PS), poly(ethylene-co-vinyl acetate) (EVA), poly(methyl methacrylate) (PMMA), polyethylene (PE), polypropylene (PP) and a commercial acrylate adhesive tape. Stable ZnO nanoparticle coatings were obtained when the polymer substrate was soluble in toluene, namely PS, EVA and the acrylate adhesive tape. In contrast, the particles were easily washed off the substrates that were not soluble in toluene (PMMA, PE, PP; see Table 1). 

This result can be attributed to the fact that the surface of PS, EVA and acrylate adhesive tape is partly dissolved by the toluene during the spin-coating process and thus becomes soft and sticky. This allows the nanoparticles to adhere to the surface better than in the case of toluene-insoluble polymers (PE, PP, PMMA), where the surface properties of the substrates are much less affected by the toluene. 

**Figure 2:**
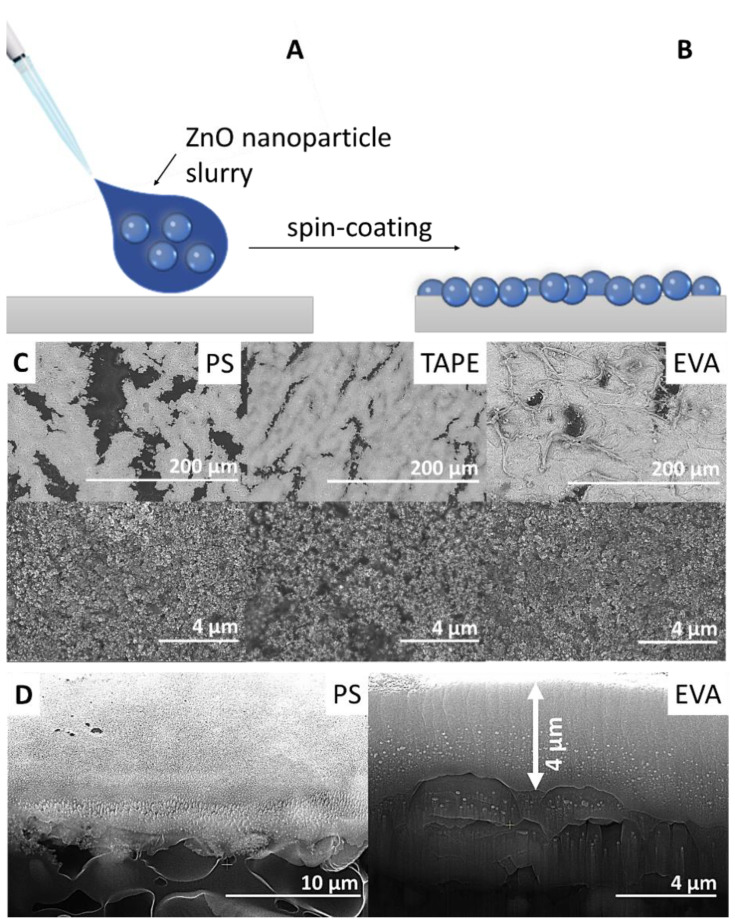
ZnO nanoparticles coating scheme: (**A**) ZnO nanoparticles slurry spin-coated onto the polymer surface, (**B**) obtained ZnO nanoparticles coating, (**C**) SEM images of ZnO nanoparticles coatings on polystyrene (PS), commercial acrylate tape and poly(ethylene-co-vinyl acetate) (EVA). (**D**) Cross-sectional SEM images of ZnO nanoparticles coatings on PS and EVA.

SEM images of toluene soluble polymer substrates coated with ZnO nanoparticles after one cycle are presented in Figure 2C. Spin-coated nanoparticles are uniformly distributed and form an almost continuous film on the polymer substrate with some pores and cracks. These defects are only in the coating layer and not in the polymer substrate itself and might be a result of the drying process. Furthermore, it is worth noting that a much less quantity of nanoparticles is needed for nearly complete surface coverage as compared to other modification techniques such as the preparation of polymer nanoparticle composites [12]. The ZnO nanoparticles are not covered by the polymer matrix (Figure 2D) and are readily accessible to the ambient environment without any post-treatment, which is vital for providing high antibacterial activity.

### 2.2. Antibacterial Efficacy of ZnO Nanoparticle Coatings

The antibacterial efficacy of the ZnO coated samples was investigated with *E. coli* (Gram-negative) and *S. aureus* (Gram-positive) bacteria. It is assumed that the polymer substrates have no effect on cell viability. Therefore, the studies listed below were performed using only coated tape samples. 

**Figure 3:**
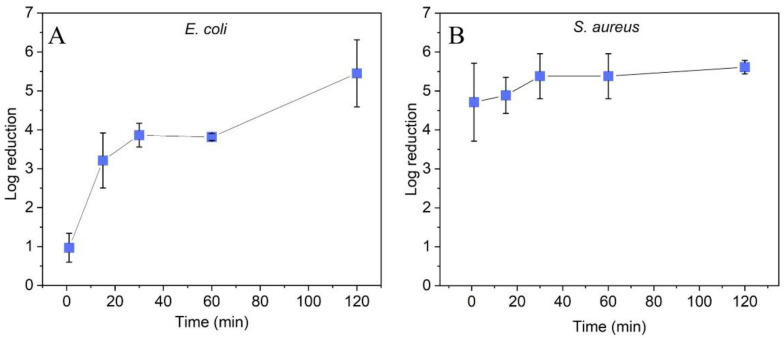
Effect of contact time to ZnO nanoparticle coated adhesive tape surfaces (single ZnO layer) on log reduction of (**A**) *E. coli* and (**B**) *S. aureus*. Standard deviation represents the average from three separate experiments.

The Gram-negative bacteria *E. coli* was more resistant to the ZnO coating than the Gram-positive bacteria *S. aureus*. After 15 min of treatment, a reduction of >3 log was observed, which did not increase significantly in the following 45 min. After 2 h of incubation, the reduction increased >5 log (Figure 3A). For *S. aureus*, a reduction of >4 log was achieved after only 1 min of contact, which further increased to >5 log after 30 min of contact time (Figure 3B). The observed differences in efficacy are consistent with previously reported observations with ZnO nanoparticles [27,28,29] and can be explained by differences in the cell membrane and accessibility to proteins and DNA [29,30]. Comparative tests with uncoated polymer surfaces showed no antibacterial efficacy for both *E. coli* and *S. aureus* (<0.5 log cell reduction over 2 h), indicating an evident impact of ZnO coating on cell viability. The antibacterial activity of ZnO nanoparticles has been explained in the literature by different mechanisms of action. It has been suggested that Zn^2+^ ions, either released into the cell culture or upon contact of the cells on the ZnO nanoparticle surface, act as a cytotoxin. Mechanical destruction or damage of the cell membrane upon contact of the bacteria with the ZnO nanoparticles has been proposed as another mechanism for antibacterial activity. The generation of reactive oxygen species (ROS), even in the dark, is discussed as a third mechanism [8,9,31].

**Figure 4:**
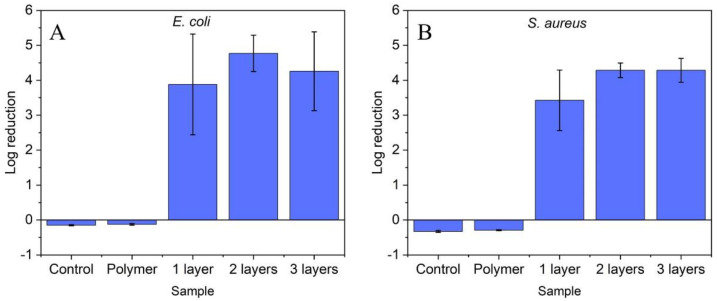
Log reduction of (**A**) *E. coli* and (**B**) *S. aureus* on ZnO coated surfaces after one, two and three deposition steps. Non-coated polymer surfaces of the same size were used as control. Standard deviation represents the average of three separate experiments.

Since SEM of single layer coated polymer substrates showed some imperfections in the coating layer (see Figure 2), the effect of deposited ZnO layer number on the antibacterial activity was investigated in the second series of experiments. The tests were again carried out with adhesive tape as substrate. The results are shown in Figure 4. While the control samples (bacterial culture only (Control) and uncoated polymer substrate (Polymer)) did not show any effect on bacterial growth, significant antibacterial activity was registered for the coated samples. However, the activity is independent of the number of ZnO layers for both *E. coli* and *S. aureus* (*p* > 0.05). On the one hand, the results can be explained by the fact that the polymer surface is already highly covered with ZnO nanoparticles after one coating step and this coverage is sufficient for the high antibacterial activity. On the other hand, it has to be considered that the high coverage of the first layer makes further stable deposition of ZnO nanoparticles on the polymer surface difficult. Consequently, only a small amount of ZnO nanoparticles can be deposited during the further coating steps. In any case, the experiments showed that already a single coating step is sufficient to obtain polymer surfaces with high antibacterial activity.

**Figure 5:**
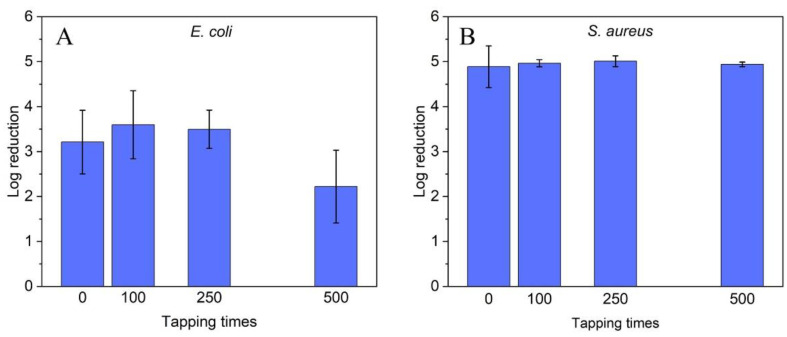
Antibacterial efficacy of ZnO coated surfaces after extensive use against (**A**) *E. coli* and (**B**) *S. aureus*. Standard deviation represents the average of three individual experiments.

The long-term stability (mechanical) and long-lasting antibacterial effect of the coatings (wear resistance) are essential prerequisites for the practical application of the coated polymer substrates. For an initial assessment of these properties, a ZnO coated substrate (adhesive tape) was touched 100, 250 and 500 times with a rubber-gloved hand. Since, when we need feedback from a surface, we always perform a sliding motion by touching a surface. Subsequently, the antibacterial properties of these samples were determined with *E. coli* and *S. aureus*. The results are summarized in Figure 5. As noted in the previous experiments, the antibacterial efficacy, expressed in log reduction, is lower against *E. coli* (initial: 3.21) than against *S. aureus* (start: 4.89). However, the antibacterial efficacy of the ZnO particle-coated samples against both *E. coli* and *S. aureus* is still present after 500 contacts. This long-term efficacy is more pronounced for *S. aureus* than for *E. coli*. For *S. aureus*, in contrast to *E. coli* (start: 3.21; final 2.22), no significant change in log reduction (start: 4.89; final 4.94) was observed. In any case, these experiments confirm the robustness of the prepared coatings.

## 3. Materials and Methods

### 3.1. Materials

Zinc oxide nanoparticles (ZnO, 20 nm) are a product of GetNanoMaterials (France). Toluene (C_6_H_5_CH_3_, 99.8%), dodecylbenzenesulfonic acid (DBSA, 95%), polystyrene (PS), poly(ethylene-co-vinyl acetate) (EVA, vinyl acetate content 40 wt.%), poly(methyl methacrylate) (PMMA) and 4′,6-diamidino-2-phenylindole (DAPI) were obtained from Sigma Aldrich (Schnelldorf, Germany). Tryptone soy broth and peptone were obtained from Oxoid Ltd., (Basingstoke, UK). All chemicals were used as received. Polypropylene (PP), high density polyethylene (HDPE, HTA108) and a commercial acrylate tape (Tesa) are products from Plastika 1 (Riga, Latvia), Exxon Mobil (Machelen, Belgium) and Beiersdorf (Hamburg, Germany), respectively.

### 3.2. Samples Preparation

Along with the commercial acrylate adhesive tape, various polymer substrates with different solubilities in toluene, such as polystyrene (PS), poly(ethylene-co-vinyl acetate) (EVA), polypropylene (PP), high density polyethylene (HDPE) and poly(methyl methacrylate) (PMMA) were prepared by hot pressing and used for deposition of ZnO nanoparticles. For the preparation of flexible polymer substrates by hot pressing, the appropriate amount of polymer was weighed to allow the preparation of 70 × 100 mm polymer films. A metal frame was used to ensure a uniform thickness of 100 μm. The temperature in the press was set according to the melting temperature of the respective polymer. The polymer was pre-heated for one minute at atmospheric pressure prior to pressing at 5 MPa pressure for 3 min. After pressing, the polymer substrates were removed from the hot press and cooled to room temperature. 

For deposition, commercial ZnO nanoparticles were suspended in toluene at a concentration of 0.3 g/mL (the highest concentration we could apply for obtaining uniform coatings by spin-coating) by using an ultrasonic probe for 2 min (200 W, Hielcher UPS200). DBSA surfactant (30 µL/mL) was added to the ZnO nanoparticles slurry to increase the suspension stability. 200 µL of ZnO nanoparticle slurry was deposited on a 5 × 5 cm^2^ polymer substrate and distributed over the surface using the spin-coating technique (rotating speed 1000 rpm). The resulting samples were heated at 40 °C for 30 min.

### 3.3. Characterization

The morphology of ZnO nanoparticles and coatings was characterized by scanning electron microscopy (SEM, Hitachi TM3000, Japan) and a high-resolution FEI Nova NanoSEM 650 field emission scanning electron microscope (FESEM, The Netherlands). The crystalline phase composition of the commercial ZnO nanoparticles was analyzed by powder X-ray diffraction (XRD, Rigaku Ultima+ diffractometer, Japan) with Cu-Kα radiation.

### 3.4. Antibacterial Efficacy Assay

Gram-positive and Gram-negative model organisms commonly used in standard protocols were selected for evaluation of the antibacterial efficacy of ZnO nanoparticle coatings. Bacterial suspensions of *Escherichia coli* ATCC 8739 and *Staphylococcus aureus* ATCC 6538 (~10^7^ cells/mL) were prepared by overnight cultivation in tryptone-soy broth. Cell numbers were estimated by direct microscopic counting. For this purpose, cells were immobilized on membranes with a pore size of 0.2 µm (Whatmann, Cytiva, Tokyo, Japan), stained with DAPI and visualized/counted by epifluorescence microscopy (excitation: 365 nm, emission > 397 nm, Axioscope 5, Zeiss). 

To determine the antibacterial properties, all samples (uncoated controls and ZnO coated) were placed in sterile containers with the treated surface facing up. A total of 200 µL of each bacterial suspension was applied to the surfaces of the samples, which were then covered with a sterile glass slide to ensure uniform and thin coverage of the bacterial suspension with good contact between the bacteria and the tested surface. After incubation periods of 1, 15, 30, 60 and 120 min in the dark, cells were detached from the surface by adding 9800 µL of 0.1% peptone, followed by vortexing for 30 s. A total of 0.1 mL of the sample was collected in at least 2 replicates, and 10-fold serial dilutions of each replicate were prepared. The respective samples were inoculated onto tryptone-soy agar plates and incubated at 37 °C for 24 h. Finally, colonies were counted and the number was estimated as CFU per ml taking the dilution steps into account. 

The effects of intensive use on the antibacterial properties of the ZnO coated samples were simulated by tapping the samples 100, 250 and 500 times with sterile powder-free nitrile gloved hands. Afterwards, the antibacterial properties were tested as described above.

All tests were performed in triplicates, each time including two technical repetitions. To exclude the influence of the natural death of the bacteria as well as of the polymer substrate itself on the antibacterial properties of the ZnO coated samples, the bacterial cultures were characterized before and after the experiments and uncoated polymer substrates (surface controls) were included in the investigations.

## 4. Conclusions

Antibacterial coatings were deposited on polymer substrates using a rapid one-step process (spin-coating at room temperature) from highly concentrated nanoparticle slurries. Robust coatings with a high coverage rate were obtained when the polymer substrate was soluble in the solvent used for slurry preparation. If this is not the case, the nanoparticles can be easily washed off the substrate surface. The ZnO nanoparticle coatings show high antibacterial activity without post-treatment of the coated substrates with a reduction of *E. coli* and *S. aureus* by more than 5 log in 120 min. High antibacterial activity was also observed after mechanical stress, highlighting the stability of the coating. Thus, using a suitable substrate solvent combination, the proposed approach can be used to apply coatings of different particles on different polymer substrates, which has not been possible so far.

## Figures and Tables

**Table 1:** Properties of ZnO nanoparticle coatings deposited on different polymeric substrates.

Polymer Substrate	Solubility in Toluene	Coating Properties
PS	+	durable
EVA	+	durable
PMMA	−	easy to remove
PP	−	easy to remove
HDPE	−	easy to remove
Acrylate tape	+	durable

## Data Availability

The data presented in this study are available on request from the corresponding author.
